# The Gut-Brain-Immune Axis in Autism Spectrum Disorders: A State-of-Art Report

**DOI:** 10.3389/fpsyt.2021.755171

**Published:** 2022-02-03

**Authors:** Chiara Puricelli, Roberta Rolla, Luca Gigliotti, Elena Boggio, Eleonora Beltrami, Umberto Dianzani, Roberto Keller

**Affiliations:** ^1^Department of Health Sciences, Università del Piemonte Orientale, Novara, Italy; ^2^Clinical Biochemistry Laboratory, Ospedale Maggiore della Carità, Novara, Italy; ^3^Mental Health Department, Adult Autism Center, ASL Città di Torino, Turin, Italy

**Keywords:** microbiota, dysbiosis, autism spectrum disorder, gut-brain axis, neuroinflammation, fecal microbiota transplantation (FMT), probiotics

## Abstract

The interest elicited by the large microbial population colonizing the human gut has ancient origins and has gone through a long evolution during history. However, it is only in the last decades that the introduction of high-throughput technologies has allowed to broaden this research field and to disentangle the numerous implications that gut microbiota has in health and disease. This comprehensive ecosystem, constituted mainly by bacteria but also by fungi, parasites, and viruses, is proven to be involved in several physiological and pathological processes that transcend the intestinal homeostasis and are deeply intertwined with apparently unrelated body systems, such as the immune and the nervous ones. In this regard, a novel speculation is the relationship between the intestinal microbial flora and the pathogenesis of some neurological and neurodevelopmental disorders, including the clinical entities defined under the umbrella term of autism spectrum disorders. The bidirectional interplay has led researchers to coin the term gut-brain-immune system axis, subverting the theory of the brain as an immune-privileged site and underscoring the importance of this reciprocal influence already from fetal life and especially during the pre- and post-natal neurodevelopmental process. This revolutionary theory has also unveiled the possibility to modify the gut microbiota as a way to treat and even to prevent different kinds of pathologies. In this sense, some attempts have been made, ranging from probiotic administration to fecal microbiota transplantation, with promising results that need further elaboration. This state-of-art report will describe the main aspects regarding the human gut microbiome and its specific role in the pathogenesis of autism and its related disorders, with a final discussion on the therapeutic and preventive strategies aiming at creating a healthy intestinal microbial environment, as well as their safety and ethical implications.

## Introduction

The human body is colonized by trillions of microorganisms accomplishing fundamental physiological and defensive functions. Together with the skin, the gastrointestinal tract (henceforth the GI tract) serves as the first interface between the host and the external environment, and it ensures a key protection against potential exogenous challenges. The GI tract can be viewed as a tripartite ecosystem, consisting of a mucosal barrier, which selectively allows the passage of cells and molecules, the microbiota, and the “second brain” (1), i.e., a neuroendocrine network to which the GI tract, the endocrine system, and the peripheral and enteric nervous systems contribute all together. According to the most widely accepted definition, the microbiota is described as “*the ecological community of commensal, symbiotic, and pathogenic microorganisms that share our body space*” (2) or as microorganisms exploiting the availability of intestinal nutrients without causing harm (3) and in some cases being beneficial to the host, a relationship that is more similar to a symbiosis (4).

The human gut microbiome consists of at least 1,800 genera and 15,000–36,000 species of bacteria, 99% of which fall within 4 main divisions: *Firmicutes* (Gram-positive aerobic and anaerobic bacteria dominated by *Clostridium* XIV and IV groups, *Lactobacillus* and *Ruminococcus*), *Bacteroidetes* (Gram-negative genera including *Bacteroides* and *Prevotella*), *Proteobacteria* (like *Escherichia coli* and other *Enterobacteriaceae*), and *Actinobacteria* (e.g., *Bifidobacterium*) (5–7). Their distribution increases progressively in a craniocaudal fashion, starting already in the oral cavity and reaching the highest density in the colon (8). Even though sampling difficulties have restrained studies on the small intestine, a molecular analysis reported enrichment of *Streptococcaceae* and *Lactobacillales* species, with reciprocal decreases in *Clostridia* and *Bacteroidetes* (5). Microbiota heterogeneity is substantially increased by the contribution given by viruses (virome), including bacteriophages (phagome), fungi (mycobiome), and parasites (parasitome), but most data available to date are on bacteria (9) (Table 1).

**Table 1:** Characterization of the most relevant microbial families along the gastrointestinal tract.

	**pH**	**Concentration (CFU/ml)**	**Microbial families**
Oral cavity	6.5–7.5	10^3^	*Candidaceae*
			*Carnobacteriaceae*
			*Fusobacteriaceae*
			*Micrococcaceae*
			*Neisseriaceae*
			*Pasteurellaceae*
			*Prevotellaceae*
			*Streptococcaceae*
			*Veillonellaceae*
Esophagus	5–7	10^1^-10^3^	*Prevotellaceae*
			*Streptococcaceae*
			*Veillonellaceae*
Stomach	1–3	10^1^-10^3^	*Enterobacteriaceae*
			*Lactobacillaceae*
			*Micrococcaceae*
			*Prevotellaceae*
			*Staphylococcaceae*
			*Streptococcaceae*
			*Veillonellaceae*
Small intestine	6–7	10^1^-10^8^	*Actinomycetaceae*
			*Bacteroidaceae*
			*Bifidobacteriaceae*
			*Candidaceae*
			*Clostridiaceae*
			*Diphteroids*
			*Enterobacteriaceae*
			*Enterococcaceae*
			*Fusobacteriaceae*
			*Lactobacillaceae*
			*Moraxellaceae*
			*Pasteurellaceae*
			*Pseudomonadaceae*
			*Staphylococcaceae*
			*Streptococcaceae*
			*Veillonellaceae*
Large intestine	7	10^10^-10^12^	*Bacteroidaceae*
			*Bifidobacteriaceae*
			*Candidaceae*
			*Clostridiaceae*
			*Enterobacteriaceae*
			*Enterococcaceae*
			*Eubacteriaceae*
			*Fusobacteriaceae*
			*Lactobacillaceae*
			*Pasteurellaceae*
			*Peptostreptococcaceae*
			*Prevotellaceae*
			*Protozoa*
			*Ruminococcaceae*
			*Streptococcaceae*

*The main microbial families are listed here (in alphabetical order) according to their distribution along the gastrointestinal (GI) tract, with differences in terms of microbial concentration (shown in the third column) and in relation to the pH of the different GI areas (displayed in the second column).*

*CFU, colony-forming units*.

It has been estimated that the non-redundant genetic content of the intestinal microbial communities outnumbers the human genome by about 150-fold, with the genetic material of bacteria and *Archaea* making up to 99% of the total (10). In addition to the “minimal gut genome,” accomplishing the basic functions of microbes, there are also microbic genes involved in the homeostasis of the entire intestinal environment and even of distant and apparently unrelated systems. Collectively, they form the “minimal gut metagenome” (10) or “holobiome,” a term that stresses the symbiotic interaction between the host and microbes (11), which has completely subverted their traditional and rather reductionist consideration as distinct entities.

The co-evolution of bacteria and humans has made bacterial biochemical functions deeply intertwined with those of the host, spanning from energy metabolism to host immunity or neurotransmitter regulation. To provide just a few examples, nearly 50% of microbial genes actively expressed are involved in the transport and metabolism of carbohydrates, amino acids, nucleic acids, and lipids, as well as in energy production, implying the enhancement of the host metabolic potential (12). Gut bacteria have a role in the fermentation of polysaccharides to short-chain fatty acids (SCFAs), such as butyrate, propionate, and acetate, later used by the host as energy sources (13), but also involved in trophic functions (14) and downstream signaling pathways with immunomodulatory (15) and tumor-suppressing effects (16). The maturation of the gut-associated lymphoid tissue (GALT) is partially dependent on the gut microbiota, which stimulates innate immunity and primes adaptive immune cells (9). Several drugs are metabolized or activated by gut bacteria, which are also able to mediate their enterohepatic circulation (17, 18). In addition, gut bacteria provide many essential amino acids (19) and vitamins like cobalamin (B12), biotin (B6), or vitamin K (20).

Paradoxically, the constant stimulation of the immune system by the gut microbiota through microbe-associated molecular patterns (MAMPs) causes a chronic “low-grade inflammation,” which is an efficacious defense mechanism, in that it keeps the immune system active and always ready to intervene. Moreover, the “beneficial” intestinal flora competitively restricts pathogen survival and proliferation, by metabolizing and consuming nutrients on one side and by producing inhibitory molecules on the other (21).

In contrast, there is also evidence supporting a potential pathogenic role of the gut microbiota in several diseases involving not only the gut, such as inflammatory bowel disease (IBD) (22), but many apparently unrelated distant body systems, such as autoimmune diseases, metabolic disorders and even neurological, psychiatric, and behavioral disorders, ranging from neurodegenerative diseases to schizophrenia and autism spectrum disorders (ASD) (8). This state-of-art report will provide a general overview of the very peculiar gut-brain-immune system axis and of the factors shaping its structure, and it will focus especially on its causative role in the pathogenesis of autism and other behavioral disorders.

## The Microbiota-Brain-Immune System Axis

### Against the Immune Privilege Theory: Recent Evidence About Immune Functions in the CNS

**Figure 1:**
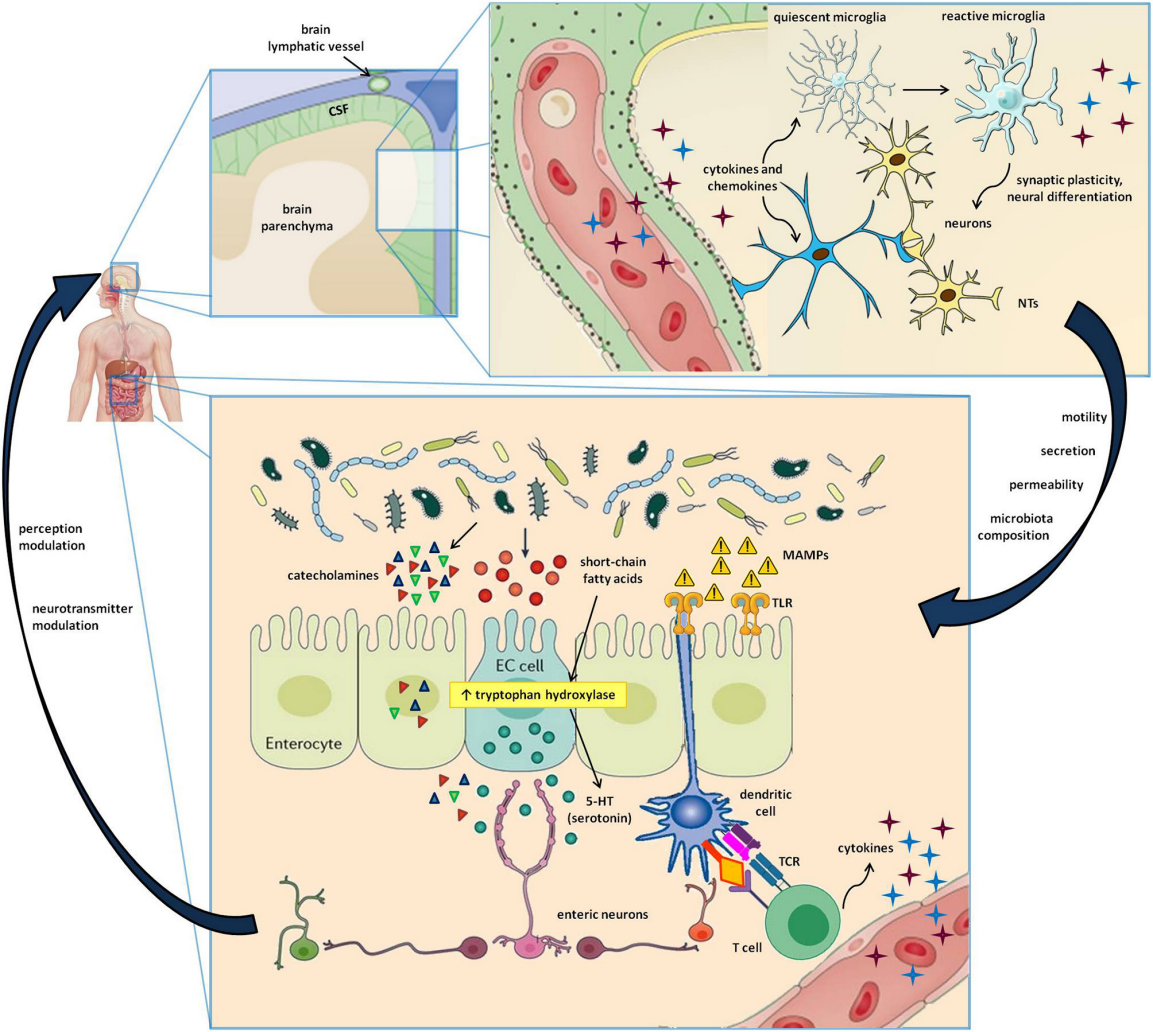
Cells and molecules involved in the gut-brain-immune axis. The lower box represents the intestinal microenvironment, where microbial niches interact with the gut mucosa by directly secreting active metabolites or by indirectly stimulating the release of neurotransmitters by enteroendocrine cells (EC cells). Furthermore, the microbe-associated molecular patters (MAMPs) stimulate Toll-like receptors (TLR) on enterocytes and dendritic cells, resulting in innate and adaptive immune triggers. The upper box shows a magnification of the cerebral parenchima—blood-brain barrier interface, where neurons, astrocytes, microglial cells, and immune cells carried by brain lymphatics all take part in a well-coordinated interplay. In addition to the signals provided by circulating molecules acting in a paracrine and endocrine fashion, the bidirectional gut-brain interaction includes afferent and efferent neural pathways also involving the autonomous and the enteric nervous systems, resulting in modulation of perception, gut motility, secretion, mucosal permeability, and even changes in the microbial ecosystem.

The brain, and more generally the entire central nervous system (CNS), has long been considered an “immune sanctuary,” with peculiar characteristics that determine its privileged status: the blood-brain barrier (BBB), which restricts the entry of peripheral immune cells, an anti-inflammatory environment, the absence of antigen-presenting and adaptive immune activity, and the lack of lymphatic tissue (23). Recent evidence has proven this dogma to be false (24). Indeed, immune surveillance in the brain is under the control of an intertwined network of both peripheral blood cells, which get access through BBB discontinuities (e.g., perivascular and choroid plexus macrophages), and resident cells that, despite not strictly belonging to the immune system, are involved in inflammation and immune defense (25). Cytokines are typically found in the CNS, where they can regulate neuronal differentiation, axonal pathfinding, and synaptic pruning in addition to their immune function (26). The same is true for class I major histocompatibility complex (MHC I) expression, which can modulate synaptic plasticity besides its antigen-presenting activity (27). The strong link between the nervous and the immune system is bidirectional. Indeed, several leukocyte subtypes release neurotransmitters affecting the polarization of neurons, while another pathway of CNS communication with the periphery is the recently described brain lymphatic system (28, 29), which, together with the BBB, facilitates the transfer of immune cells and molecules into and out of the brain (28–30) (Figure 1).

One of the best models to describe the neuroimmune interaction is probably the microglia. This CNS cell population of erythromyeloid origin not only plays a role in the immune surveillance of the brain (25), but it also influences synaptic pruning and neuronal circuit wiring and plasticity during CNS development (31) (see below). Microglial cells have a macrophage-like behavior, in that they are provided with class II MHC (MHC II) proteins to present antigens, display phagocytic activity, and release inflammatory cytokines and chemokines (23). However, their function is controversial, since their final role depends on the signals received by other immune cells, such as T lymphocytes and nervous cells, such as astrocytes. While patrolling the CNS, microglia swings between a pro-inflammatory (M1) and an anti-inflammatory (M2) phenotype depending on the current needs, thus ensuring a proper immune balance (32).

The CNS homeostatic balance is also strongly dependent on astrocytes, which are crucial to BBB integrity, cerebral blood flow, nutrient supply (being the main site of glycogen storage), neurotransmitter turnover, and synapse formation (33). In addition, despite their non-hematopoietic origin, they play a role in the modulation of neuroinflammatory responses (34). Astrocytes usually downregulate microglial inflammatory activity, but the microglial pro-inflammatory M1 phenotype predominates when enough pro-inflammatory signals block the astrocyte-mediated inhibition (32).

### Neurotransmitter Modulation by the Gut Microbiota

Unexpectedly, the gut microbiota is potentially implicated also in neurophysiology, involving many pathways connecting the gut and the peripheral and central nervous systems and providing evidence for a bidirectional relationship named “gut-brain axis” (35). Indeed, there are several ways in which the gut microbiota influences the nervous system and even human behavior and *vice versa*.

First, it is involved in neurotransmitter modulation through direct and indirect production of signaling molecules such as serotonin, dopamine, γ-aminobutyric acid (GABA), histamine, and acetylcholine (36). Almost 90–95% of circulating serotonin is released predominantly by gut enterochromaffin cells (EC cells) (37). Their activity may be regulated by gut bacteria through the production of small molecules (such as SCFAs) that potentiate the secretion of these neurotransmitters by EC cells by inducing the expression of tryptophan hydroxylase (38).

Second, several gut bacteria directly synthesize key neurotransmitters, which are decreased in germ-free animals (39). Through afferent pathways, these neurotransmitters modulate CNS activity and may even modulate the perception of several stimuli. This phenomenon may explain the mechanism underlying antibiotic-induced visceral hypersensitivity, which is attenuated by the administration of probiotics to restore the usual commensals (40). Since these molecules play a role also in the regulation of higher cognitive functions, such as reward and motivation, mood, stress responses, attention, and learning, an intriguing hypothesis suggests that dysbiosis, i.e., an alteration of the common microbial intestinal environment, might be a cause of neurological, mental, and behavioral disorders (8, 35).

Third, the CNS itself may influence the composition of the gut microbiota and gut permeability, both directly, through the modulation of GI motility and mucosal secretion, and indirectly, via signaling molecules released by EC cells, immune cells, and neurons, such as vasoactive intestinal peptide (VIP), serotonin, melatonin, GABA, catecholamines, histamine, and acetylcholine (9). In fact, the gut microbiota expresses several neurotransmitter receptors, which accounts for a direct effect of the CNS on the gut flora (41). All these tasks are accomplished in concert with the autonomic, enteric, and neuroendocrine systems, with a contribution coming also from the hypothalamus-pituitary-adrenal axis (41, 42) (Figure 1).

### The Role of the Intestinal Microbiota in the Modulation of Neuroinflammation and CNS Injury

Given the premises that intestinal microorganisms can influence the immune response and that the CNS is no more considered an immune-privileged site, it follows that the gut microbiota likely takes part in the CNS-immune system intercommunication. A first interaction is exemplified by microbial metabolites of dietary tryptophan, such as the indole-3-aldehyde of *Lactobacillus reuteri*, which can become ligands of astrocytic aryl hydrocarbon receptors (AHR), attenuating inflammation (43, 44). In a study by Rothhammer et al. depletion of tryptophan-metabolizing bacteria using ampicillin in mice worsened the severity of experimental autoimmune encephalitis (EAE), the animal model of multiple sclerosis (MS), while subsequent supplementation with tryptophan metabolites improved EAE recovery (43). Additionally, the gut microbiota may indirectly affect CNS inflammation by modulating peripheral immune cells. *Bacteroides fragilis* capsular polysaccharide A (PSA) and other bacterial metabolites, including SCFAs, can induce IL-10-producing regulatory T cells (T_reg_) and can inhibit differentiation of inflammatory T helper type 1 and 17 (T_h_1 and T_h_17) lymphocytes (45, 46). Conversely, segmented filamentous bacteria behave as T_h_17 inducers (47), reproducing the typical inflammatory pattern of EAE (48). This might be of therapeutic importance in MS and other autoimmune diseases (47, 49, 50).

Besides the role in CNS immunomodulation, the microbiota is also directly involved in CNS injury. Mouse models showed that treatment with amoxicillin and clavulanate reduced post-ischemic infarct volumes (51), but treatment with ciprofloxacin and metronidazole increased damage (52). Neuroprotection was related to reduced infiltration of small intestinal IL-17-producing γδ T lymphocytes (51), implying that intestinal bacteria influence immune cell trafficking to the brain with differential effects depending on the microbial species, which explains the opposite effect of different antibiotic therapies.

Overall, there is now a growing body of evidence supporting a strong implication of intestinal microorganisms in the gut-brain-immune system loop, where they behave as a double-edged sword, with both neuroprotective and pro-inflammatory effects (Figure 1).

## The Gut-Brain Axis in Prenatal Life

### Sterile Womb Dogma or *in utero* Hypothesis? Theories About Early Exposure to the Microbiota

There is still a lot of debate about the timing of bacterial intestinal colonization. The traditional “sterile womb” paradigm, according to which bacterial colonization starts during and immediately after birth during vaginal delivery (vertical transmission) and through contact with the surrounding environment soon thereafter (horizontal transmission) (53–55), has been recently challenged by the detection of bacterial DNA in the placenta, amniotic fluid, and meconium (56). Still, the newly coined “*in-utero* colonization hypothesis” (57) must be taken with caution, since the use of highly sensitive molecular techniques may lead to false-positive results. The genetic material detected could derive from dead microorganisms or contaminating bacteria, a chance also supported by the proven ability to raise sterile experimental animals after Cesarean section (C-section) (55).

Postnatal colonization is a consolidated theory and even the type of delivery would determine which microbial strains will predominate. Vaginal delivery exposes the newborn to the maternal gut and perineal microorganisms, such as *Bacteroides, Bifidobacterium*, and *Lactobacillus*, while a C-section leads to delayed colonization predominantly by skin bacteria like *Streptococcus* and *Staphylococcus* (58).

Nevertheless, the fetus is inarguably exposed at least to the products of the maternal microbiota during gestation, such as microbial metabolites and cell wall components crossing the placental barrier (59, 60). Translocation of gut bacteria from the intestinal lumen to the maternal bloodstream is increased during pregnancy and lactation (61), through mechanisms likely involving dendritic cells (62), goblet cells (63), or microfold cells (64). Overall, this results in a facilitated placental transfer of microbial molecules to the fetus (61), which contributes to shaping the immune system, metabolic control, and even behavioral aspects. For instance, PSA from maternal *B. fragilis* contributes to the generation of T_reg_ cells in the fetal gut mucosa, improving tolerance to dietary antigens and protecting against inflammation (65). Furthermore, several studies support the relationship between reduced early colonization by the intestinal flora and the risk of developing autoimmune and immune-mediated diseases like arthritis (66), type 1 diabetes (8, 67), asthma (68), IBD (69), and allergies.

In turn, the maternal microbiota composition depends on several factors, such as diet, antibiotic exposure, and other environmental influences. An experiment on primates confirmed the role of a maternal high-fat diet (MHFD) in shaping the intestinal microbiome of the offspring (70). Besides, there is evidence that maternal consumption of probiotics correlates with a reduced risk for preterm delivery (71) and development of allergic diseases in the offspring (72, 73). Conversely, antibiotic treatment may restrict or alter fetal exposure to bacteria and their products, resulting in aberrant immune priming. In mice, treatment with antibiotics during pregnancy decreased IL-17-producing cells and production of granulocyte colony-stimulating factor (G-CSF) and increased the risk for neonatal sepsis (74). This suggests that the effect of intestinal bacteria on the immune response consists in an overall modulation aimed at maintaining a proper balance between pro-inflammatory and anti-inflammatory states mediated by T_h_17 and T_reg_ cells, respectively (75).

**Figure 2:**
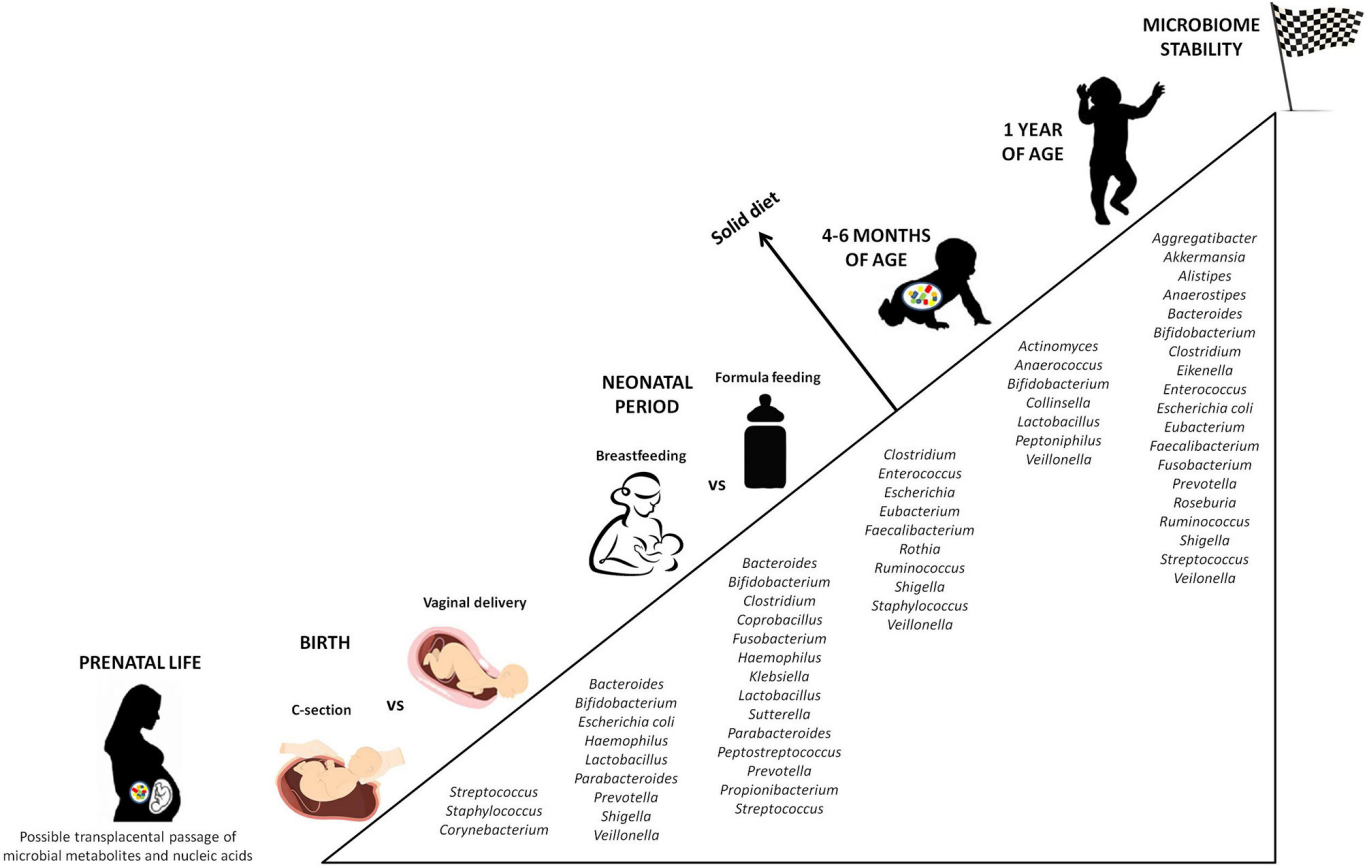
Progressive colonization of the gut mucosa by bacterial species. The figure displays the major microbial species colonizing the intestine from prenatal life to early childhood, highlighting the differences according to external factors able to influence the microbial composition (C-section vs. vaginal delivery, breastfeeding vs. formula feeding). Stability of the microbial ecosystem is reached around 1 year of age, when the child microbiota has become similar to the maternal one. An additional influencing role is played by the diet in the first months of life. Not surprisingly, the introduction of solid food causes a significant change in the intestinal microbial composition.

Later in life, antibiotic therapies, infections, nutritional habits (breast-feeding vs. formula feeding), stress, and genetics are postnatal factors shaping the intestinal microbial community (76). In fact, the infant microbiota constantly evolves, until a phase of stability is reached between 6 and 36 months of life. In this time-lapse, the gut flora can be divided into a constant endogenous and autochthonous group (the core microbiome) and a provisional, highly resilient one (the allochthonous microbiome), sensitive to exogenous stimuli (77). While the newborn gut is dominated mainly by *Bifidobacterium, Enterococcus, Escherichia, Shigella, Streptococcus*, and *Bacteroides*, by the age of one it is inhabited by *Clostridium, Ruminococcus, Alistipes, Eubacterium*, and *Prevotella*, much closer to the maternal microbiota, and more anaerobic (78) (Figure 2).

Finally, the impact of maternal microbiota and early exposure to environmental microorganisms is both age-dependent and tissue-specific. Previous reports showed that the formation of lymphoid tissue in the lung and the promotion of T_reg_-mediated immune tolerance are influenced by the exposure to endotoxins and bacteria in the first 2 weeks of life, but not during adulthood (79, 80). The final results depend on the type of microorganism the newborn comes into contact with (66). Overall, this implies that the microbiota-driven pathogenesis of immune-related diseases acts in critical windows of times, which must be taken into account when planning prevention strategies acting on microbiota (66).

### Microbiota-Driven Modulation of Nervous System Development

Evidence for the implication of microbiota in nervous system development derives from studies showing that germ-free mice exhibit hyperactivity, increased risk-taking behaviors, reduced anxiety, and learning and memory impairment (81, 82), similar to some behavioral and cognitive disorders of children. Moreover, alterations in the expression levels of serotonin receptors (5-HT_1A_), NMDA glutamate receptors, and neurotrophic factors like brain-derived neurotrophic factor (BDNF) have been detected in the hippocampus, dentate gyrus, and amygdala of these mice (81–84), together with a dysfunctional BBB (85) and a more myelinated prefrontal cortex (86).

Nervous system development starts around the third week of gestation in humans, when neural stem cells originating from the ectoderm begin to differentiate into a thick, pseudostratified neuroectoderm, or neural plate, which forms in a craniocaudal fashion mimicking the spatial organization of the rest of the body. This structure undergoes differential expansion and folding to give rise to the future brain and spinal cord. Moreover, its lateral tips contain neural crest cells that detach and migrate to many body locations, including the gut (87). In addition to a massive neuronal expansion, a constant reshaping of neural pathways takes place throughout the whole gestational period, so that about 50% of neurons will undergo apoptosis by the end of pregnancy (88). Exposure to neurotrophins like BDNF is crucial in determining the fate of neurons: the more synaptic connections are created, the higher the concentration of neurotrophins surrounding the neuron and fostering its survival (89). Germ-free mouse models display a significant alteration of synaptic plasticity, with increased neurogenesis in the hippocampus and amygdala and a consequent increase in their volumes (84, 90). It seems therefore that early exposure to intestinal bacteria or their metabolites keeps excessive neuronal proliferation under control and favors pruning of unconsolidated synapses. Of notice, postnatal colonization of germ-free mice does not reverse this phenotype, which reinforces the theory that there is an optimal timing for the nervous system to be influenced by the gut microbiota (90).

In contrast, postnatal life is dominated in the first two or three decades by gliogenesis, a crucial process for the regulation of synaptic plasticity and pruning and for the development of higher cognitive functions (88). Unlike neurons, astrocytes, and oligodendrocytes, microglia consists of CNS-resident immune cells derived from yolk-sac erythromyeloid progenitors and involved in immune surveillance and tissue repair. Moreover, microglial cells release a wide variety of cytokines and chemokines that drive neuronal differentiation, synaptic circuit wiring, and pruning, a process that goes under the umbrella term of synaptic plasticity (91). Decreased maturity of microglial cells in the white and gray matter have been detected in both germ-free mice and after antibiotic treatment. These developmental defects could be rescued by the administration of SCFAs, which are key metabolites released by intestinal bacteria (92). In addition, microglial gene expression does not depend on the general bacterial load but on specific bacterial taxa, so that some species are more able than others to cause abnormalities in microglia. The precise mechanisms explaining this phenomenon have not been defined yet, but probably involve indirect SCFA-mediated signaling pathways (92).

The absence of gut microorganisms also increases the permeability of the BBB by decreasing the expression of the tight junction proteins occludin and claudin-5 (85). Together with the recently described lymphatic vasculature of the brain (28, 29), a permeable BBB favors the passage of immune cells and metabolites into and out of the brain, making it more vulnerable to external factors.

Through these mechanisms, the maternal gut microbiota may be involved in the pathogenesis of some neurological and behavioral disorders. Neurogenesis is also controlled by an interplay between the immune system and the gut microbiota. Treatment with probiotics is associated with the expansion of CNS monocytes, whose depletion reduces neurogenesis, suggesting that these circulating innate immune cells can modulate the microbiota-gut-brain communication (35).

## The Role of the Gut-Brain Axis in Neuropsychiatric and Behavioral Disorders

### Hypotheses About the Microbiota-Driven Pathogenesis of Mental Disorders

If the gut microbiota is so deeply involved in the regulation of many physiological functions extending beyond the GI tract, the hypothesis that it might have a role also in shaping human behavior is probably not so surprising. There is a constantly growing body of scientific literature dealing with the implication of the gut-brain axis in the pathogenesis of mental disorders, to such an extent that supporting evidence has been found even in much less complex organisms like the fly *Drosophila*, often used as an experimental model to explain fundamental biological mechanisms (93–95).

Several studies in mice have outlined a link between microbiota-driven neuroinflammation, neurotransmitter modulation, and altered behavioral outcomes. A possible model to describe the pathogenesis of behavioral disorders is the maternal immune activation (MIA) animal model. A study by Hsiao et al. demonstrated that if the maternal immune system is intentionally activated during pregnancy, the offspring shows behavioral abnormalities similar to those found in ASDs. Interestingly, when the researchers analyzed the gut microbiota and intestinal mucosa of the offspring, they found an altered microbial flora and an increased intestinal permeability. However, oral treatment with the probiotic *B. fragilis* during weaning could ameliorate both the dysbiosis and the behavioral abnormalities (96). MIA is also associated with elevated levels of circulating IL-6 and IL-17A (97), involved in MIA-induced T_h_17 cell differentiation. Since some intestinal microbial species are known to downregulate pro-inflammatory T_h_17 cell differentiation and, thus, CNS inflammation, it follows that the gut microbiota must be entailed not only in the multifactorial etiology of cognitive and behavioral disorders but also in the protection from them.

Regarding neurotransmitter modulation, it has been found that MHFD, known to modify the gut microbiota (70), may result in abnormal social behavior in the offspring, probably due to reduced hypothalamic expression of oxytocin and reduced activity of the reward system dominated by dopamine (98). Furthermore, Bravo et al. demonstrated that supplementation with *Lactobacillus rhamnosus* could alter GABA receptor expression in the cortical regions, hippocampus, and amygdala, resulting in reduced anxiety- and depression-related behaviors and decreased stress-induced corticosterone levels, suggesting the involvement of the neuroendocrine axis. Interestingly, these effects were abrogated after vagotomy, implying a fundamental role of the peripheral nervous system in the gut-brain connection (99).

**Figure 3:**
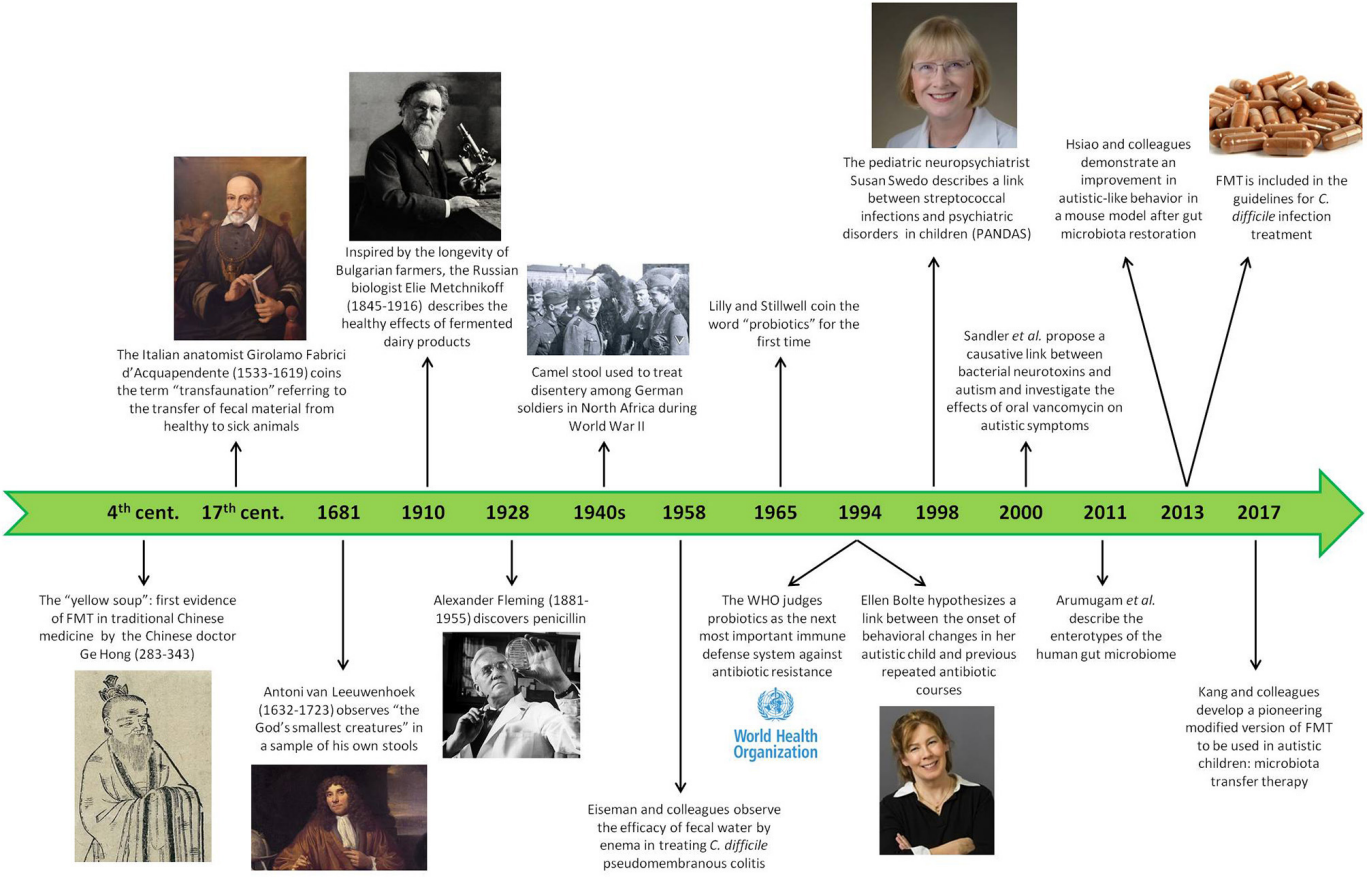
History of the gut microbiota. This timeline shows the most significant facts about the attitude, the first trials, and the scientific goals in the field of intestinal microbiota during human history, starting from ancient times until nowadays.

These concepts can be applied to human behavior, too, even though the research in this field is still at its onset. Already in the 1990s, the pediatric neuropsychiatrist Susan Swedo was able to demonstrate a link between bacteria and psychiatric disorders. She described a subgroup of young children with obsessive-compulsive and tic disorders characterized by a peculiar explosive onset, greater symptom severity, and relapsing-remitting course, and she noticed a correlation with previous streptococcal infections. This intuition led her to coin the new definition of pediatric autoimmune neuropsychiatric disorders associated with streptococcal infection (PANDAS) (100–102), a first step in the research about the causative role of microorganisms in neuropsychiatric disorders (Figure 3).

## Focus on Autism Spectrum Disorder

### Overview and First Evidence of Microbiota Implications in Autism Spectrum Disorder

Autism spectrum disorder (ASD) is a neurodevelopmental disorder with an early onset and a genetic component. ASD is characterized by deficits in socio-emotional reciprocity, impaired verbal and non-verbal communication skills and difficulties in developing and maintaining adequate social relationships with peers, associated with the presence of repetitive verbal and motor behaviors, restricted patterns of interest, need for an unchanging (or in any case predictable and stable) environment, and hypo- or hypersensitivity to sensory inputs. The onset is in the early developmental period, but the first manifestations could become apparent when the child starts to face social demands (around school-age or slightly before) (103). The last epidemiological updates report a prevalence of 1 in 44 children (104). ASD should be supported lifetime in most cases, also in adulthood (105).

There is a constantly evolving debate about the pathogenesis of this multifaceted behavioral disorder, which seems to depend on several individual and external factors (106). Far from being related to a single mendelian mutation, the genetic background is rather complex and includes more than 100 genes mainly involved in CNS development, as well as an even greater number of polymorphisms conferring susceptibility. Moreover, ASD may be one of the manifestations of monogenic syndromes, such as Fragile-X syndrome, Rett syndrome, and neurofibromatosis (NF) (107). Proposed environmental factors include infections, often associated with molecular mimicry and immune-mediated pathogenesis, immune system impairment, allergies, nutritional deficiencies or overload, and errors during embryonic neural tube closure (6). There is also evidence of transient brain overgrowth in autistic children (108), which might be directly linked to an altered synaptic scaffolding and epigenetic modulation of synaptic plasticity (109). The process of synaptic pruning is believed to be aberrant in ASD, resulting in an excessive number of synapses, probably also due to a dysregulated microglial function (110). In any case, focusing on a single causative agent would be extremely reductive for a so complex and variable group of disorders, and the genetic dysregulation could be also linked to other neurodevelopmental disorders, schizophrenia, and bipolar disorder (111–113).

The impact of the gut-brain axis on animal behavior has raised interest in studying a possible implication of the intestinal microbiota in the pathogenesis of autism and related disorders. This hypothesis has been also suggested by the observation of a higher prevalence of GI comorbidities in autistic children (114, 115), which also strongly correlates with the severity of their behavioral symptoms (116). Besides, autistic subjects display an altered permeability of the gut mucosa and consequently higher levels of endotoxins (117) and oxidative stress biomarkers (116) in their serum compared with neurotypical children. An inverse correlation with socialization scores has been demonstrated also in this case (117).

Investigation of the qualitative composition of fecal samples in autistic children has allowed to characterize a different bacterial taxonomy compared to neurotypical children, with an increased presence of *Bacteroidetes* at the expense of *Firmicutes* (except *Clostridium*, which is increased), and an altered colonization by *Bifidobacter, Lactobacillus, Prevotella, and Ruminococcus genera* (118–120). Even though usually weakly represented, *Candida albicans* is twice more abundant in autistic toddlers, and it can release ammonia and other toxins (121).

Altogether, these findings have made the hypothesis of microbiota involvement in autism more than a simple speculation and have paved the way for interventional studies trying to disentangle the complex pathogenesis of this disorder. Several mechanisms might explain the microbiota-driven pathophysiology of ASD, mostly connected to dysbiosis. The following section will provide an explanation for this apparently reductive concept.

### Pathophysiology of ASD: The Microbiota Point of View

The alteration of the intestinal microbial composition can shift the balance from a commensal microbial community to a potentially pathogenic ecosystem. It has been postulated that the decreased levels of *Bifidobacterium* may deprive the gut of its protective anti-inflammatory activity, while the relative abundance of the SCFA-producing *Bacteroidetes* may be deleterious for the gut-brain axis (6).

Colonization by *Clostridium* species is increased, too, and the first to hypothesize its possible role in the pathogenesis of autism in 1998 was Ellen Bolte, mother of an autistic child who understood the link between the onset of her child's behavioral changes and previous repeated antibiotic courses causing chronic and likely *Clostridium*-related diarrhea. This led to the speculation of a subacute, chronic *Clostridium tetani* infection as a cause of the neurodevelopmental disorder (122). Later on, Sandler et al. (123) proposed the contribution of bacterial neurotoxins to autistic symptoms and undertook a pioneering trial investigating the effect on behavioral and communicative difficulties of an 8-week treatment with oral vancomycin, minimally absorbed by the GI mucosa and thus specifically targeting the gut lumen and its flora. Despite the small dimension of the sample group (only 11 children) and the short-term improvement registered, probably owing to antibiotic-resistant *Clostridium* spores (123), the results were promising and indicated that the research in this field was going in the right direction (Figure 3).

Dysbiosis also induces the so-called “leaky gut” phenotype, i.e., an increase of intestinal mucosa permeability (120). Interestingly, ASD children have higher plasma levels of zonulin, a protein modulating gut permeability, and its concentration correlates with symptom severity (124). A leakier mucosa facilitates inter-epithelial and trans-epithelial passage of bacteria and their products, which are thus able to reach the circulation and directly affect the CNS with neurotoxic signals (e.g., MAMPs, LPS, exotoxins, SCFA, neuropeptides).

In this regard, the advent of metabolomics in the last few years has allowed to effectively identify alterations in the metabolites of autistic subjects. It has been estimated that about 90% of autistic children have peculiar dietary preferences, especially for starches, snacks, and processed foods, whereas they mostly reject fruit and vegetables (125). Dietary components are the main external modulators of the gut flora (126), which in turn can affect the metabolic profile by taking part in digestive processes. Not surprisingly, urinary, fecal, and serum samples from autistic children have been shown to contain low levels of antioxidants (127), high levels of bacteria-derived phenolic compounds, and a high concentration of SCFAs (acetic acid, propionic acid, but not butyric acid), products of undigested carbohydrates which may influence the CNS by modulating catecholaminergic biosynthesis (120, 128, 129), altering mitochondrial function in neurons, and epigenetically modulating the expression of ASD-related genes (120, 130). The levels of free amino acids (FAAs) are much higher in autistic children compared to controls, and they correlate with an increase of proteolytic bacteria like *Clostridium* and *Bacteroides* (120). Worth mentioning is glutamate, whose increase might play a role in the etiopathogenesis of neurodevelopmental disorders (131), leading to excitotoxicity and neuronal cell death (120).

Additionally, a significant contribution derives from the “inflammation hypothesis” (6), according to which ASD correlates with a chronic inflammatory state and immune dysfunction (124). As a matter of fact, the transcriptional profile of ileal and colonic tissues of autistic subjects overlaps significantly with the one of IBD patients (132). Besides, examination of intestinal biopsies from autistic children has revealed infiltration of inflammatory cells mimicking IBD and food allergies (133), as well as an excess accumulation of advanced glycation end products (AGEs) believed to induce neuroinflammation and neurodegeneration (134). Owing to a disrupted gut mucosa, the CNS is highly exposed also to pro-inflammatory cytokines, such as interleukin (IL)-1β, IL-6, IL-8, IL-12, tumor necrosis factor (TNF)-α, and transforming growth factor (TGF)-β (135), which overcome their confinement to the gut and promote CNS inflammation and alteration of neuronal signaling.

Neurotransmitter alteration is an additional potential mechanism linking dysbiosis with ASD. In addition to the potentially toxic effect of glutamate mentioned above, evidence of hyperserotonemia in the blood of autistic children has been provided since the 1970s (136). Conversely, hyposerotonemia has been reported in the brain of autistic subjects (137). Because 90% of circulating serotonin derives from intestinal EC cells (37), some authors claim that the alteration of this biomarker may be of intestinal origin and, even more, might be related to dysbiosis. According to DeTheije, the low-grade intestinal inflammatory state of ASD children may stimulate EC cells, mast cells, and platelets to synthesize serotonin, inducing intestinal dysmotility and consumption of tryptophan (138). At the same time, dysbiosis can reduce the availability of tryptophan by decreasing dietary amino acid absorption (139). As a result, despite the higher levels of circulating serotonin originating from the gut, the effective availability of this neurotransmitter to the CNS may be actually reduced. Unfortunately, neither tryptophan supplementation nor selective serotonin reuptake inhibitors (SSRIs) have ever been demonstrated to be effective (6).

Finally, the intestinal microbiota influences the development and maturation of both the immune system, which leads back to the theory of neuroinflammation, and the nervous system, with a special impact on neurogenesis, pruning, and modulation of synaptic connections. In this sense, the theory supporting the pathogenic role of an excessive number of synaptic contacts due to glial functional derangement and altered synaptic selection can be further supported.

These results can be considered revolutionary, in that they add another piece to the complex and multifactorial pathogenesis of these disorders. Although there is still little consensus on the exact role of the gut microbiota in ASD, and despite the opposing results of some studies, a new perspective has been opened in the field of neurophysiology, with potentially revolutionary future therapeutic implications.

## Microbiota-Oriented Therapeutic Strategies

### The Yellow Soup: A Brief Historical Perspective on Gut Microbiota Manipulation

Even though the emergence of scientific interest in gut microbiota is quite recent, the knowledge of gut bacteria role and importance in human health has been known for centuries. References to sour milk or fermented foods date back even to the Bible and, already in fourth century China, one of the treatments for diarrhea or food poisoning implied the use of the so-called “yellow soup,” also known with the more pretentious name of “golden syrup,” which consisted of fecal material from healthy subjects (140, 141). A similar habit was described among African Beduins, used to consuming the feces of their camels to fight dysentery (141), a practice which would be used hundreds of years later by the German soldiers of the African troops to recover from dysentery in the early 1940s (142). In the fifteenth century, the Italian anatomist Acquapendente coined the concept of “transfaunation” to refer to the transfer of fecal material from healthy to sick animals, thus founding a new therapeutic approach also in veterinary medicine (141), while the “God's smallest creatures” observed by Antoni van Leeuwenhoek (1632–1723) in his own feces provided real evidence that microorganisms could be detected in human stools (141). The largest contribution to the field of intestinal microbiota was probably given by the Russian Nobel prize Metchnikoff (1845–1916), who went beyond the simple use of fecal matter to treat some gastrointestinal disorders by observing (and experiencing on himself) the impact of daily consumption of fermented dairy products on improved health and longevity in Bulgarian peasants, thus pointing out the role of fecal microbiota also in prevention strategies (143). However, it was only in 1965 that the word “probiotics” was first introduced by Lilly and Stillwell (144) and later better defined by Parker as “live microbial supplements which beneficially affect the host animal by improving its microbial balance” (145).

Starting from the second half of the nineteenth century, interest in the field of gut microbiota has grown exponentially, transcending its mere application to gastrointestinal disorders and exploring its involvement in several physiological and pathological body processes, ranging from metabolic control to cardiovascular health, and even to the recently emerged concept of the “gut-brain axis” as a basis for many neurological and mental disorders. The international recognition came in 1994 when the World Health Organization (WHO) judged probiotics to be the next most important immune defense system in case of resistance toward antibiotics (146), and in 2013 fecal microbiota transplantation (FMT) was included in the guidelines for *Clostridium difficile* infection for the first time (147) (Figure 3).

### Dietary Interventions

Modifications of the diet have become a popular non-pharmacological attempt to improve ASD symptoms. Disordered nutritional habits are quite common among autistic children, with a prevalence exceeding 50%, and it seems that their food selectivity is not just a matter of taste and routine reflecting the typical repetitive and restrictive behavior, but it would derive also from an altered sensory perception, another key clinical finding in ASD, which affects several sensory domains and determines a hyperresponsiveness to food taste as well. In turn, frequent food refusal and scarce variety in the food repertoire, with a preference for sweet, carbohydrate-rich, and fatty foods and a paucity of fruit and vegetables, might imply metabolic disorders and deficiencies of essential nutrients including vitamins like vitamin C, D, and B12, minerals like calcium or zinc, and essential aminoacids (148). Besides, ASD children, or at least a subgroup of them, often have an altered bowel function and decreased digestive enzyme activity, which probably accounts for the elevated urinary levels of dietary peptides and inflammatory putrefactive metabolites (i.e., propionic acid) in fecal samples (149).

According to the “opioid excess theory” and to the “leaky gut theory,” metabolic derivatives of protein digestion, especially casein and gluten, may easily penetrate through a highly permeable intestinal barrier and act as agonists of CNS opioid receptors, negatively affecting neurotransmission and worsening ASD symptoms (150). Conversely, a fiber-rich diet seems to be essential to maintain a healthy and diverse gut bacterial ecosystem (151). Dietary fibers mainly consist of microbiota-accessible carbohydrates (MACs), i.e., monosaccharides behaving as primary energy sources for the gut flora and contributing to the creation of ecologically different niches according to their chemical variety (151).

Based on these premises, some attempts were made to act on the diet as a potential non-evidence-based therapeutic intervention to improve symptoms in autistic children. The first promising results were shown by some prospective studies revealing a positive effect of gluten- and casein-free diets especially on behavioral symptoms, attention, communication, and hyperactivity of ASD, later corroborated by randomized clinical trials with a control group as a comparison (152–155).

However, the enthusiasm generated by these results is counterbalanced by the methodological flaws that investigators may encounter, which are primarily due to the small sample size, short study duration, lack of blinding, participant dropout, reporting bias linked to a parental placebo effect, and especially to poor patient compliance. In this regard, a very recent meta-analysis by Keller et al. (156) actually concluded for very little evidence in favor of a gluten- and casein-free diet, while two randomized clinical trials were unable to find a demonstration against or in favor of gluten and casein avoidance, also because the statistical significance of the inter-group differences is highly dependent on patient adherence and on the scoring system used to evaluate the impact on autistic symptoms, which frequently suffers from subjectivity (157, 158).

Moreover, dietary changes have been inarguably associated with potentially adverse consequences and there are concerns that they would be difficult to implement in the child's daily life. In a disorder characterized by very peculiar dietary preferences and a picky eating behavior (149), restrictive diets would further limit food variety, increase social withdrawal and lead to macro- and micronutrient deficiencies (159) and to weight loss (156). The sponsored gluten- or casein-free diets do not seem to restore the microbiota, they often have little fiber and protein content (160), and since they often result in an increased intake of simple, refined carbohydrates to replace gluten-containing foods, they may actually promote toxic bacteria overgrowth (149).

In addition to dietary restrictions, some trials evaluated the effect of specific nutritional supplements, including vitamin D and the antioxidant phytochemical sulphoraphane. A meta-analysis by Li et al. (161) demonstrated a significant but still small improvement in hyperactivity after cholecalciferol supplementation for a period ranging from 3 to 12 months, but without any appreciable benefit on other key autistic symptoms. Conversely, the results obtained by a randomized placebo-controlled trial where participants received sulphoraphane for 18 weeks were more promising, leading to improved social interactions and verbal skills in the treatment group. However, the effect was limited to the intervention period since the clinical scores returned to pretreatment levels short after discontinuation (162).

On the whole, despite the solid theoretical explanation of this intervention, there is still no robust evidence in favor of a specific diet with the aim to ameliorate autistic symptoms, or at least this approach should be personalized on an individual basis.

### Prebiotics and Probiotics

Given the possible involvement of dysbiosis in ASD, attempts have been made to modify the microbiota composition in order to shift the balance from a potentially pathogenic to a possibly beneficial microbial community or, at least, to restore eubiosis, using prebiotics and/or probiotics.

Prebiotics are non-digestible compounds that, when metabolized, support the proliferation of beneficial gut bacteria (6), such as the bifidogenic galacto-oligosaccharides (163). Despite the interest in these dietary supplements, recent researches mostly focused on probiotics, i.e., living non-pathogenic microorganisms displaying beneficial effects in GI and extraintestinal disorders including IBS, IBD, *Clostridium*-related diarrhea, colorectal cancer, and neurological diseases (6). The combination of prebiotics and probiotics in the same therapeutic strategy allows to obtain an even more beneficial synergistic effect; these combinations have been defined as *symbiotics* (164).

Evidence supporting the use of probiotics dates back to the already cited elegant experiment by Hsiao and colleagues, who obtained a restoration of the gut microbiota and an improvement of autistic-like behavior in the mouse model of MIA (96), and by subsequent trials studying the effect of a probiotic course on the amelioration of ASD symptoms. Promising results were also obtained in a recent randomized placebo-controlled trial on autistic boys aged 7–15, where treatment with *Lactobacillus plantarum* for 28 days significantly ameliorated the opposition/defiance and impulsivity/hyperactivity behaviors linked with this disorder. Notably, the effect appeared to be age-dependent, with better results obtained in younger children, underlining the importance of early intervention (165).

In this regard, the enthusiasm generated by probiotics has transcended their mere therapeutic application and has prompted an attempt to use them also in prevention strategies. A randomized clinical trial was performed on 75 infants, who were split into two groups to receive either *L. rhamnosus* or a placebo for the first 6 months of life. Surprisingly, after a 13-year follow-up, 6 out of 35 patients in the placebo group were diagnosed with neurodevelopmental disorders like Asperger syndrome (AS) and attention-deficit/hyperactivity disorder (ADHD), while none of the children in the probiotic group had developed these conditions (166). This exciting result supported an epigenetic role of microbiota and showed a promise of the efficacy of probiotic administration in the attenuation of ASD symptoms.

Nevertheless, a number of unresolved questions remain open, and additional validated randomized controlled trials are needed to further support this therapeutic proposal. In fact, most studies carried out so far have several limitations, such as the heterogeneity of patients and problems of safety, tolerability, compliance evaluation, and of a proper validation (8, 120, 149). Moreover, one of the most frequently used methods to assess symptomatic changes is the Autism Treatment Evaluation Checklist (ATEC), a caregiver-administered questionnaire to measure changes in ASD features after a treatment (167), which again raises concerns about the subjectivity and the reporting bias affecting the results. Only a few studies demonstrated a significant amelioration of behavioral symptoms, concentration, and sociability after a course of probiotics, with most being prospective and not randomized controlled trials, while others were able to demonstrate an impact only on GI symptoms, if any (168). This might instill the doubt that the benefit on the behavior is indirectly due to an improvement of the frequent gut symptoms encountered among children with ASD rather than to a real effect on ASD-specific manifestations. A recent suggestion is to consider ASD as consisting of at least two distinct subtypes, i.e., ASD with or without GI dysfunction and comorbidities. This dichotomy should be taken into account in the therapeutic plan of ASD children.

Overall, supporting a “one for all” therapeutic approach would be reductive. The deep heterogeneity of ASD phenotypes, in terms of severity, predominating symptoms, comorbidities, and patient personality, prompts an individualized approach, which considers all the shades of this complex behavioral disorder.

### Microbiota Transplantation

In addition to oral supplementation of probiotics, direct transplantation of eubiotic flora into the gut has attracted many researchers since Eiseman and colleagues observed its efficacy in the treatment of *C. difficile*-related pseudomembranous colitis in 1958 (169). There are two main strategies to perform this operation: fecal microbiota transplantation (FMT) and microbiota transfer therapy (MTT) (Figure 3).

FMT consists in the transfer of fecal microbiota from healthy donors to patients to restore a dysbiotic intestinal ecosystem (170). Firstly experimented in the field of gastroenterology, FMT has shown promise also in the therapy of extraintestinal disorders, including metabolic syndrome (171), multiple sclerosis (172), Parkinson's disease (173), neurodevelopmental disorders like ASD (174), and even cancer (175, 176). The procedure implies the collection of at least 50 g of donor feces, which should be processed within 6–8 h. Alternatively, a frozen aliquot stored in stool banks is thawed to room temperature. The stool sample is then suspended with non-bacteriostatic saline, water, or milk to obtain a 200–500 ml injectable suspension, and filtered to remove large particulate matter (177). All donors are screened before donation and excluded in case of recent antibiotic treatment, immunosuppressive therapies, GI disorders, and other risk factors for infection transmission (178). Finally, after a proper bowel preparation of the recipient, the fecal material is transferred via the upper GI route (through an oral capsule, esophagogastroduodenoscopy [EGDS], or nasogastric, nasojejunal, or nasoduodenal tubes) or via the lower GI route (through colonoscopy or retention enema) (178). The latter is usually preferred since it carries a lower risk of aspiration into the airways (179).

MTT has been developed as an evolution of FMT when Kang and colleagues developed a pioneering modified protocol to be applied to 18 ASD children, consisting of 14 days of oral vancomycin, a day of fasting bowel cleansing, and 7–8 weeks of oral or rectal administration of standardized human gut microbiota (SHGM). At the end of this open-label trial, significant improvements were observed in ASD symptoms, especially the behavioral ones (180). Notably, these benefits were maintained and even improved after a 2-year follow-up of these subjects (181), suggesting the long-term effect this therapeutic strategy could have.

Autologous transplantation is another tested option, for instance to restore the microbial environment after antibiotic treatment or before surgery to prevent post-surgical GI sequelae (182). Unlike drugs, the autologous microbiota transplant is not foreign to the body, but it is part of a complex ecosystem, whose composition is simply modified after the procedure. These “natural” properties of FMT have made it appealing to both clinicians and patients and account for the raising interest of the last few years.

However, only a few studies on human subjects have been designed as clinical trials so far, and most of them have been mainly observational or case reports. Validated interventional studies are strongly awaited in the near future, but the evidence accumulated in the last few years has highlighted the therapeutic potential of these new treatment modalities and how they could positively modify patient outcomes.

### Safety Issues Regarding Microbiota Transplantation

This state-of-art report has provided a lens to see the advantages and the potential benefits derived from FMT and gut microbiota manipulation in several fields of medicine. Nevertheless, these practices are not devoid of possible dangers and adverse events, which must be taken into account when planning a therapy using this approach. So far, only a few FMT-associated deaths have been reported, mainly caused by aspiration pneumonia after midgut FMT (179, 183, 184) or by toxic megacolon (185). However, the possibility of contaminated microbes in the donor feces and the FMT procedure itself may cause serious adverse events, which has led some medical teams to change their protocols after experiencing these criticalities, for example performing midgut FMT only in the awake patient to prevent aspiration (179) or including the possibility of catastrophic colitis, sepsis or death in the FMT consent form to be safeguarded (185).

Legal issues are an additional concern and difficulties have emerged also regarding the definition of microbiota transplantation, since its classification (whether or not it can be considered a drug or whether it should be regarded as a human tissue) is the starting point for the regulation of industrial, stool banks and insurance companies policies. Despite some overlap, FMT cannot be considered a probiotic product, since probiotics do not produce a durable engraftment as FMT and they are grown in pure cultures, decreasing the risks of transferring pathogens to the patient. Nor it can be regarded as a typical drug, since most drugs have a single specific target, while transplanted microbiota have an impact on the entire intestinal ecosystem and even beyond. Moreover, their effectiveness is difficult to test in animals, which makes the typical drug regulatory pathways problematic, and each batch is different from the others, complicating the characterization of transplanted material. After some debate, a National Institute of Health (NIH)-funded working group reached a consensus in 2015, according to which microbiota transplantation was defined as “the transfer of biologic material containing a minimally manipulated (i.e., not altering the original relevant characteristics) community of microorganisms from a human donor to a human recipient (including autologous use) with the intent of affecting the microbiota of the recipient” (182).

On the whole, the future perspectives regarding gut microbiota applications in medicine imply also an attempt to improve the policies regulating its applications and especially to introduce new regulatory schemes to reach an international harmonization.

## Conclusions

This state-of-art-report outlined the multifaceted relationship between the gut microbiota and the brain, which has been hard to demonstrate for a long time, despite the supporting hypotheses (141). The recent introduction of high throughput technologies has surely provided great help to carry on the investigations into this intriguing field, allowing the detection of key metabolites, neurotransmitters, receptors, and signaling pathways that were inconceivable just a few decades ago. The take-home message could be summarized with three key concepts.

First, the Cartesian assumption of the human body as a machine, divided into clearly distinct and individually functioning organ systems and a clear mind-body dualism has been overcome by the most recent findings (186, 187). It would be better to view our organism as a complex cluster of constantly intercommunicating organs, whose functions almost always extend beyond their anatomical location to influence other apparently unrelated targets. In other words, a real ecosystem. The gut microbiota represents a perfect example of this new concept since it consists of live microorganisms that, despite being exogenous, become a fundamental symbiotic component of the human body, forming a real organ and contributing to its evolution.

Second, in the field of medicine, the gut-brain axis theory has enabled us to take several steps forward in the comprehension of the pathophysiology behind several intestinal and extra-intestinal disorders, including neurodevelopmental ones like ASD. New unexpected pathways have been described, and some previously unquestionable dogmas have been subverted, with an even more revolutionary impact on therapeutic strategies.

Third, in line with the scientific trend of the last few years, patient therapy should not be considered as a “one-fits-all” approach. The future perspective aims at personalizing treatments as much as possible, to take all the patient needs and individual variability into account. In this regard, the research in the field of gut microbiota and the gut-brain axis has given a fundamental contribution.

## Limitations

The primary goal of the authors was to provide a state-of-art report about the relationship between the gut-brain-immune system axis and the pathophysiology of ASD. As such, this work is not intended to be a systematic review or a meta-analysis containing statistical data in favor of or against these pathogenic models and the therapeutic interventions inspired by them. Moreover, no, strict inclusion or exclusion criteria were used to select the articles used as sources of information.

## Author Contributions

CP, LG, EBo, and EBe: bibliographic search and selection. CP and RR: writing—original draft preparation. UD and RK: writing—supervision, review, and editing. UD and EBo: funding acquisition. All authors contributed to the article and approved the submitted version.

## Funding

This work was supported by IG 20714 Associazione Italiana per la Ricerca sul Cancro, Milan, Italy; Fondazione Cariplo (2017–0535). Fondazione Umberto Veronesi, Milan, Italy supports EBo.

## Conflict of Interest

The authors declare that the research was conducted in the absence of any commercial or financial relationships that could be construed as a potential conflict of interest.

## Publisher's Note

All claims expressed in this article are solely those of the authors and do not necessarily represent those of their affiliated organizations, or those of the publisher, the editors and the reviewers. Any product that may be evaluated in this article, or claim that may be made by its manufacturer, is not guaranteed or endorsed by the publisher.
